# Effect of the land area elevation on the collective choice in ants

**DOI:** 10.1038/s41598-017-08592-9

**Published:** 2017-08-18

**Authors:** Olivier Bles, Nathanaël Lozet, Jean-Christophe de Biseau, Alexandre Campo, Jean-Louis Deneubourg

**Affiliations:** 1 Center for Nonlinear Phenomena and Complex Systems (Cenoli) - CP 231, Université libre de Bruxelles (ULB), Campus Plaine, Boulevard du Triomphe, Building NO - level 5, B-1050 Bruxelles, Belgium; 2 Evolutionary Biology and Ecology (EBE) - CP 160, Université libre de Bruxelles (ULB), Campus du Solbosch, 50 Avenue Franklin D, Roosevelt, B-1050 Bruxelles Belgium

## Abstract

Collective decisions regarding food source exploitation in social insects are influenced by a range of parameters, from source quality to individual preference and social information sharing. Those regarding the elevation of the physical trail towards a food source have been neglected. In this work, we investigated the effect of ascending and descending paths from the nest to a food source on collective choice in two ant species *Lasius niger* and *Myrmica rubra*. Our hypothesis that returning loaded with food from the high source is more energy efficient was validated by choice experiments: when the sources are simultaneously introduced the high food source is preferentially exploited by both species. The flexibility of colony response was then tested by introducing the preferred source (high) incidentally, after recruitment towards the down food source began. Despite the well-known lack of flexibility of *L*. *niger*, both species showed the ability to reallocate their foraging workforce towards the highest food source. The collective choice and the flexibility are based on the difference between the u-turn rates when foragers are facing the ascending or descending branch. We discuss these results in terms of species-specifics characteristics and ecological context.

## Introduction

Collective exploitation of food resources in social insects, such as ants or honeybees, is largely based on a high rate of information sharing among workers in the colony. This communication results in food recruitment, allowing quick and efficient exploitation of large food sources^1–3^, focusing their activity preferentially on the best-quality food source (for example, in *Apis mellifera* ^4^ or in *Lasius niger* ^5^), and maximizing energy efficiency^6^. Several mechanisms corresponding to positive feedbacks, occur in the phenomenon of food recruitment in social insects, from the waggle dance in honeybees to indicate the direction of a food source^7, 8^, to physical recruitment of nestmates by tandem running^9^, through pheromone deposition by foragers back and forth between a food source and the nest^10^. Other positive feedbacks are also described such as short range interactions at the food source^11^. Recruitment involving trail is the most widespread type of recruitment in ants^12^, providing strong positive and negative feedback to make decisions on colony foraging^13, 14^. It has been shown that the type of recruitment (and thus the species) affects the ability of colonies to direct their foraging activity as a function of the opportunities offered by the environment^15, 16^. For example, when a *L*. *niger* colony is confronted with two equal food sources of a 1 M sucrose solution, the colony concentrates its activity on one of the two sources or paths. If the solutions are of different quality, e.g., 1 M vs 0.1 M, the colony will select the richer food source. Indeed, a wide range of ants modulate their trail laying in relation to food source quality allowing the preferential exploitation of the most rewarding source when they are discovered simultaneously^15, 17–19^. Thus, foragers of the ants *L*. *niger*, lay 43% more trail marks when exploiting a 1 M sugar source than those exploiting a 0.1 M source^1, 5^. However, if a poor food source is already being exploited, *L*. *niger* cannot shift its foraging activity to a more rewarding source presented subsequently^15^. By contrast, *Myrmica sabuleti* and *Tetramorium caespitum* rapidly shift, even when the difference in concentration is not so great (e.g., 1 M vs 0.1 M and even 0.5 M in 30% of trials, respectively^15, 20, 21^).

It has been suggested that the mechanisms of recruitment to a food source could be the origin of this difference between *L*. *niger* or even *Myrmica* spp^22^, which practise mass recruitment, while *Tetramorium spp*. practise more leader-based recruitment^17^. Mass recruitment implies a high fidelity to a pheromone path and could lead to a sub-optimal choice if the colony has started to exploit one source while a richer one appears incidentally^15^. In contrast, prioritizing the influence of the leaders in groups that recruit towards a newly discovered food source rather than following a chemical trail gives the system a greater flexibility. Owing to their individual memory, the leaders are able to guide a group of recruits towards a new food source without paying attention to the chemical trail leading to another source. However, it has to be noted that within colonies, the individual behavioural series of foragers are quite diverse: some lay a continuous trail to the nest, some mark only some points in a dotted pattern, and others return rapidly to the nest without laying trails and perform a complex of activating actions inside the nest^23^. In addition, categorizing ants as exhibiting either “mass recruitment” or “leader-based recruitment” is an over-simplification, as a range of strategies of recruitment can be observed in a colony, for example depending on the colony size or the moment of observation during a foraging bout^22^. For example, *Tetramorium* spp shift from “leader-based recruitment” (group recruitment) to a trail recruitment when the number of foragers involved in the recruitment increases^15, 24^.

Numerous parameters influencing collective choice have been studied in social insects, including nest-source distance^25^, the quality and nature of sources^20, 26^ or even effect of geometry on path choice^27–33^ with a main focus on the maximization of foraging gains; however, there is little direct, empirical evidence to show that animals select routes that minimize costs. Indeed, the parameters that characterize landforms, the relief (used as synonymous of elevation in this paper), has been largely neglected, even though they can affect the ants’ foraging decision to exploit resources in the tree canopy, such as aphids^34^, or resources at the floor level. It is already known that leaf-cutter ants such as *Atta* and *Acromyrmex* spp., are likely to learn a new trail^35^ or adapt their load transport^36^ and walking speed^37, 38^ according to the physical characteristics of a foraging trail. While it can be equivocal^39^, it has been suggested that inclines could be more energetically costly than walking on a flat surface (See Introduction in ref. 40), even for insects^41, 42^, resulting from an increase in biomechanical constraints^43^. In a route-selection experiment using foraging wood ants, it was shown that the route was primarily determined by energetic cost (estimated indirectly from the vertical height traversed), although the journey time was also a factor^6^. Moreover, it is not common for two well-known ant species to be tested in strictly the same experimental setup, and this can be a good starting point for discussions about general or observed species-specific behaviours/strategies.

Our study aims to characterize the collective responses of *L*. *niger* and *Myrmica rubra* resulting from recruitment competition in binary choice experiments. Firstly, we focused on investigating the effect of food source accessibility, that is having to walk on descending or ascending same-length branches towards identical food sources, on the collective choice of the colony. The gross benefit of both food resources is equal, but we emphasize that the latter maximizes foraging efficiency, as gravity acts on the forager’s load^38, 44^ and makes a descending slope when travelling back to the nest easier to cross than an ascending one. Thus, the net energy intake (described as the food resource benefits minus the cost of retrieving it) of an ascending branch leading to a food source could be more profitable, and this branch should therefore be preferentially used by both *M*. *rubra* and *L*. *niger* when both food sources are simultaneously available. Undoubtedly, foraging does generally incur costs, and balancing these costs against potential losses from a path to a food source is essential for effective foraging.

Our second aim is thus to evaluate to what extent these colonies were able to not get stuck in suboptimal decisions by balancing the cost/benefit ratio: the flexibility of a foraging workforce will be tested by first introducing only the down food source (at the end of a descending branch), and the second one (at the end of an ascending branch) will be available incidentally (after recruitment to the down food source has initiated). Based on past studies about the flexibility of collective choices in both *Lasius* ^15^ and *Myrmica* ^20^, we assumed that in case of differential introduction of food sources, *L*. *niger* recruits should be trapped, despite the lower benefit, by the already established trail towards the down food source, while *M*. *rubra* should be able to reallocate their workforce towards the ascending and energetically efficient path.

## Results

### Foraging activity at the colony level and collective choice

**Figure 1:**
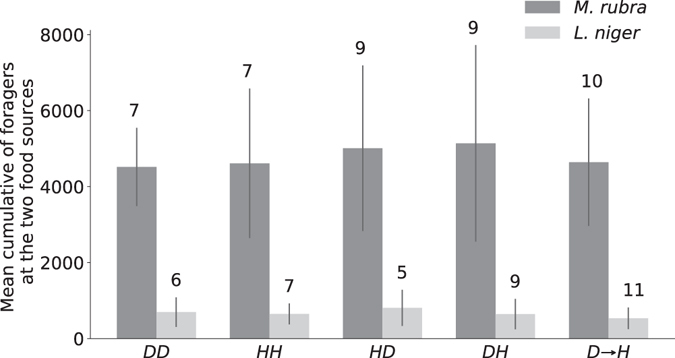
Cumulative number of foragers at the two food sources for 2 h in each condition. Mean and standard deviation; numbers above bars = number of replicates. *DD* = Down-Down, control condition, two descending paths to food sources. *HH* = High-High, control condition, two ascending paths to food sources. *HD* = High-Down, experimental condition, left path ascending to food source and right path descending to food source. *DH* = Down-High, experimental condition, left path descending to food source and right path ascending to food source. *D* → *H* = Down → High, experimental condition, only down food source available at the end of left path during the first 20 min of experiment before incidentally introduction of the high food source at the end of the right branch.

The experimental condition had no effect on the overall effort of food collection (see Data Collection and Analysis for definition); regardless of the conditions the foragers globally spent the same amount of time on food sources whether we consider the case of *M*. *rubra* (Fig. 1, KW, K = 0.20, P = 0.99) or *L*. *niger* (Fig. 1, KW, K = 2.8, P = 0.99). The cumulative number of *M*. *rubra* was approximately 7 times greater than the number of *L*. *niger* at a food source (4802 ± 2000 and 644 ± 371, respectively); this was mainly due to the four-fold longer mean time spent by a forager of *M*. *rubra* at a food source (321 ± 146 sec, N = 63) compared to *L*. *niger* (75 ± 20 sec, N = 70) (see below, individual-level behaviour section). As the cumulative number of ants at the two food sources was not different for the conditions *DH* and *HD* (See Fig. 1), for clarity, we pooled them together (*DH*) for the upcoming results of the *M*. *rubra* and *L*. *niger* experiments.

**Figure 2:**
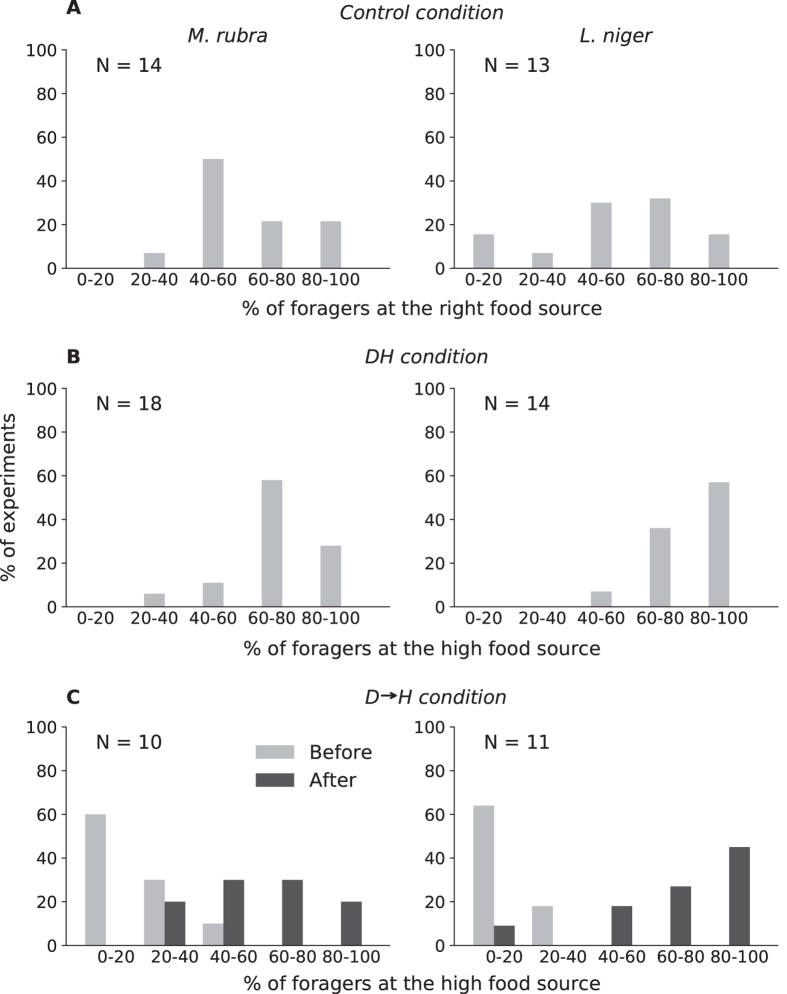
Strength of choice towards a food source. Percentage of experiments against percentage of total foragers present at the high food source for each experimental condition and species. For the *D* → *H* condition, as in Table 1, we separated the results into two phases, before and after the introduction of the second food source.

Concerning the collective choice of food source, in the control conditions (*DD* and *HH*) *M*. *rubra* showed no choice of food source in 7/14 experiments and *L*. *niger* in 4/13. A significant choice of a food source was highlighted in 7/14 experiments for *M*. *rubra* and in 9/13 replicates for *L*. *niger* but these choices were randomly distributed between left and right food source. Figure 2A represents the percentage of experiments characterized by a given % of foragers at the right food source in control conditions (*DD* and *HH* pooled). For both *M*. *rubra* and *L*. *niger*, a peak appeared at approximately 50%, suggesting that in the majority of experiments, approximately half of the total number of foragers randomly chose the right or left food source.

In the *DH* and *HD* conditions, we observed a clear preference for the high food source in both *M*. *rubra* (15/18) and *L*. *niger* (13/14) (Table 1). Approximately 60% of the experiments were characterized by more than 75% of the ants choosing the high food source (Fig. 2B).

**Table 1:** Food source preference of each experiment for both species in *DH* and *D* → *H* conditions.

Species	*DH*		*D* → *H* 20 *min*		*D* → *H* 120 *min*	
	Down	High	Down	High	Down	High
*M*. *rubra*	2/18	15/18	9/10	0/10	2/10	5/10
*L*. *niger*	0/14	13/14	9/11	0/11	1/11	8/11

Number of experiments with significant choices versus total number of experiments (binomial test with P < 0.05). In *DH* condition, both species strongly exploit preferentially the high food source. In *D* → *H*, while both species exploit the down food source until 20 min (as the high one is only available after 20 min *(D* → *H 20* *min*)), at the end of experiment, respectively 5/10 (*M*. *rubra*) and 8/11 (*L*. *niger*) colonies preferentially exploit the high food source *(D* → *H 120* *min*).

In the D → H condition, a majority of choices at the end of first 20 min (when only the down food source was introduced) were significantly oriented towards the down food source (*M*. *rubra*: 9/10, *L*. *niger*: 9/11). Of the 9/10 experiments for *M*. *rubra* and 9/11 for *L*. *niger*, 55% (5/9) and 88% (8/9) switched to the high food source after its introduction, respectively. During the first 20 min, the majority of foragers exploited the down food source (less than 50% exploiting the high food source) in all experiments, while at the end of 2 h, the majority of foragers of all colonies exploited the high food source (Fig. 2C). For both the *DD* and *HH* control conditions and the *DH* and *HD* conditions, no bias towards the left or right food source was highlighted.

### Individual-level behaviour

*M*. *rubra* showed no preference for the first discovered source in the *DH* condition (Table 2, high: 9/18, down: 8/18; in one experiment, two foragers simultaneously discovered both food sources), while *L*. *niger* preferentially discovered the high food source (11/14). The time needed to discover the first source was 132 ± 81 sec, 119 ± 66 sec and 134 ± 138 sec, respectively, in the *DH*, *HH* and *DD* conditions for *M*. *rubra* and 75 ± 70 sec, 64 ± 47 sec and 123 ± 209 sec for *L*. *niger*.

**Table 2:** First food source discovered in the *DD*, *HH* and *DH* conditions.

Species	*DD*		*HH*		*DH*	
	Left	Right	Left	Right	Down	High
*M*. *rubra*	5/7	2/7	2/7	5/7	9/18	8/18
*L*. *niger*	3/7	4/7	3/6	3/6	3/14	11/14

**Figure 3:**
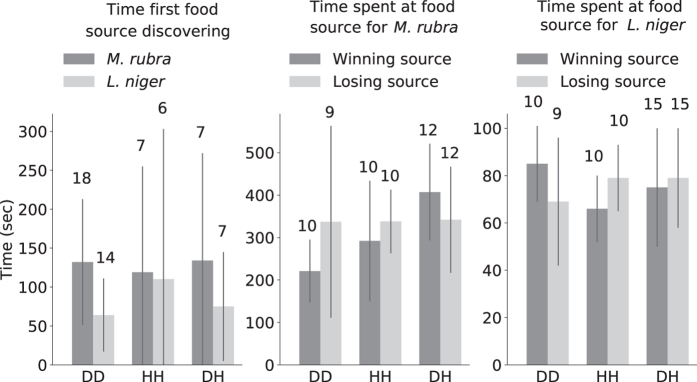
Discovery of and time spent at food sources. Mean and standard deviation; numbers above bars = N.

The experimental condition did not affect the time of first source discovery (see Fig. 3) for *M*. *rubra* (KW, K = 0.18, P = 0.91) or for *L*. *niger* (KW, K = 0.89, P = 0.64). Moreover, the delay between the discovery of the first and second food source was short, below 120 sec for approximately 80% of the experiments in both studied species. The time spent at a food source was not affected by the experimental condition in either species, regardless of focusing on the winning or losing food source (*M*. *rubra*: K = 15.39, P = 0.0088; *L*. *niger*: K = 6.60, P = 0.25).

As the preference of both species to exploit the high food source was very clear, we were interested in determining whether individuals showed different travel speeds on the ascending and descending arms, in both nest-to-food source travel and the reverse. In both species, this travel speed was very similar in all conditions (see Table 3; *L*. *niger*: between 8.1 ± 1.7 sec and 8.5 ± 1.8 sec; *M*. *rubra*: between 19.3 ± 7 sec and 23.8 ± 7.3 sec) and showed no significant difference (KW, K = 2.84; P = 0.41) among the different experimental conditions. Having established that branch slope had no effect on the travel speed of foragers, we then examined the behaviour of ants arriving at the Y-maze and facing an ascendant or descendant branch. Considering the *DH* condition, the proportion of U-turns in the exploration phase in the Nest → Source direction was significantly lower when ants faced the ascendant branch (4%; N = 157) than the descendant branch (45%; N = 94) in *L*. *niger* (Table 4; two-sample binomial test, P < 0.0001). Values were similar in *M*. *rubra*: 7% (N = 145) of U-turns towards the ascending branch and 52% (N = 73) towards the descending branch (Table 4; two-sample binomial test, P < 0.0001). During the phase of exploration in the *D* → *H* condition, the results were similar for both species: the U-turn rate was approximately 10% (N = 154) towards the ascending branch and rose to 46% (N = 140) towards the descending branch in *L*. *niger* (Table 4; two-sample binomial test, P < 0.0001), while it was 10% (N = 63) towards the ascending branch and rose to 48% (N = 80) towards the descending branch in *M*. *rubra* (Table 4; two-sample binomial test, P < 0.0001). Even more surprising were the results of the U-turns in phase of recruitment in the *D* → *H* condition: even if a pheromone trail led *L*. *niger* foragers towards the down food source for approximately 20 mins, as it was the only one available (a few moments after having introduced sucrose in the high food source), we observed an interestingly high U-turn rate on the descending branch, approximately 41% (N = 75), while it was very low and significantly different towards the ascending branch, 3% (N = 234) (Table 4; two-sample binomial test, P < 0.0001). Observations were similar for *M*. *rubra* in the *D* → *H* condition, with a U-turn rate of 41% (N = 49) towards the down food source for approximately 20 mins, while it was only approximately 3% (N = 73) towards the ascending branch. In the *DD* control condition during the exploration phase, the U-turn rate was high and not significantly different between the left and right branches in *L*. *niger*, with a U-turn rate of 68% (N = 100) towards the losing source and 67% (N = 101) towards the winning one (Table 4; two-sample binomial test, P = 1). Surprisingly, the results were very similar in the recruitment phase; the U-turn rate only slightly decreased compared with the phase of exploration and remained particularly high on the losing branch (60%; N = 67) as well as on the winning one (40%; N = 171) and did not significantly differ between the winning and losing branches (Table 4; two-sample binomial test, P = 0.057). The U-turn rate for the winning branch was significantly different between the exploration and recruitment phase (67% and 40%, respectively; two-sample binomial test, P = 0.01), while no difference appeared for the losing branch (68% and 60%, respectively; two-sample binomial test, P = 0.53). The results were similar for *M*. *rubra* during the phase of exploration, with no significant difference in the U-turn rate towards the losing branch (46%; N = 184) and the winning one (51%; N = 206) (Table 4; two-sample binomial test, P = 0.68). During the phase of recruitment, the U-turn rate differed only slightly and not significantly (two-sample binomial test, P > 0.05 for each branch for the two phases observed): 45% (N = 180) of U-turns towards the winning source and 41% (N = 162) towards the losing one, and no significant difference appeared between the two branches (Table 4; two-sample binomial test, P = 0.68). In the *HH* control condition, a very low U-turn rate was observed for both the winning and losing sources in the exploration and recruitment phases in *L*. *niger* as well as *M*. *rubra* (see Table 4).

**Table 3:** Travel speed on the ascendant and descendant branches.

	Nest → High source	Nest → Down source	Nest → High source	Nest → Down source
*L*. *niger*	8.2 ± 2 (30)	8.1 ± 1.7 (30)	8.5 ± 1.8 (30)	8.8 ± 1.4 (30)
*M*. *rubra*	19.3 ± 7 (30)	22.5 ± 6.9 (30)	21.75 ± 6.6 (30)	23.8 ± 7.3 (30)

The time (in sec) required to cross a branch was measured in the *DH* condition. Time needed to cross a branch was measured between points (2) and (3) for the ascending branch and between (4) and (5) for the descending one (see 4).

**Table 4:** U-turn rates on ascendant and descendant branches towards the food sources in different conditions.

	Exploration		Recruitment	
	High source	Down source	High source	Down source
***L***. ***niger***				
*Down* **-** *High*	4% (157)	45% (94)	—	—
*Down* **→** *High*	10% (154)	46% (140)	3% (234)	41% (75)
	**Winning source**	**Loosing source**	**Winning source**	**Loosing source**
*Down* **-** *Down*	67% (101)	68% (100)	40% (171)	60% (67)
*High* **-** *High*	3% (105)	5% (40)	3% (174)	5% (83)
*M*. *rubra*				
*Down* **-** *High*	7% (145)	52% (73)	—	—
*Down* **→** *High*	10% (63)	48% (80)	3% (73)	41% (49)
	**Winning source**	**Loosing source**	**Winning source**	**Loosing source**
*Down* **-** *Down*	51% (206)	46% (184)	45% (180)	41% (162)
*High* **-** *High*	6% (119)	7% (72)	1% (154)	0% (45)

The U-turn rate was measured in all experimental conditions for the two studied species. For the *Down-Down* and *High-High* control conditions, values were counted for both left and right sources and classified as “winning” and “losing” sources.

## Discussion

In agreement with our predictions, our experiments showed that when facing a Y-maze with one ascending and one descending branch, both leading to the same quality food source (1 M sucrose), ants of *M*. *rubra* and *L*. *niger* clearly preferred to exploit the food source at the end of the ascending branch (in approximately 90% of the experiments). Indeed, in the asymmetrical condition (*HD*), colonies of both species could easily discriminated between the two branches, focusing their foraging effort on the highest food source along the ascending slope in almost all experiments, allowing us to qualify the ascending one as the preferred path or and the descending one as the non-preferred path. We also showed that in this context, both species were able to provide evidence of flexibility, as they reallocated their foraging workforce from the descending branch to the ascending one when incidentally introduced in 72% of experiments. The highest food source was introduced 20 min after the lowest one, as preliminary work showed a maximum presence at food sources occuring approximately 20–30 min, which is and not long enough to completely fill up the food stock at the intranidal level, as foraging activity started to decline only after approximately 60 to 90 min.

The results of our investigations regarding the ability of both species to reallocate the colony’s foraging effort from a well-established trail on a non-preferred path to a new preferred path leading to a new food source are not consistent with certain results of past studies. Indeed, it has been shown that if a poor food source is already being exploited, *L*. *niger* cannot shift its foraging activity to a more rewarding source presented subsequently^15, 45^, while *M*. *sabuleti* is capable of switching, even when the difference in quality is not so great^20^. Our experiments were conducted with two food sources of equal concentrations of sucrose, with the only difference being an ascending or descending branch to a food source. The observed shift in food source for both species was quite surprising, as it revealed that a range of parameters are taken into account in collective choice in ants and that modifying only one of them (here the “slope” of the path) could radically changed the previously known collective response of ants.

It appeared that in the symmetrical conditions of *DD* and *HH*, colonies of both species were able to exploit the two sources evenly, and approximately 60% of the experiments randomly led to the choice of one source (symmetry breaking). Among the 60% of experiments, a comparison of *HH* and *DD* showed no significant difference in the level of symmetry breaking. In addition, the total amount of food retrieved by foragers was not significantly greater in *HH* than in the *DD* condition.

A past study on *L*. *niger* showed that if two equally concentrated sources of sucrose were simultaneously presented, colonies focused on one of them^15^. This strongly asymmetrical pattern of foraging among food sources is caused by symmetry breaking. The non-linearity of individual choice behaviour in response to the strength of signals for different trails determines asymmetry in foraging^46, 47^. In addition, it has been shown that the degree of non-linearity is a key element in determining the level of asymmetry exhibited by foraging social insects and can be influenced by several parameters, particularly pheromone quantity, a factor dependent on forager number (i.e., colony size)^48, 49^. It is clear that symmetry breaking can either be enhanced or mostly avoided through the modification of individual responses to recruitment stimuli and that the number of foragers changes the effects of the relative non-linearity of the choice^49^. Therefore, it has been shown that small colony size (like our experimental colonies) can caused low levels of asymmetry^48, 50^ and may facilitate the switch in paths observed in the *D* → *H* condition. Larger colony size produces a stronger asymmetry between the choices. It should also be the case in our HD experiments. However, despite this stronger asymmetry, the flexibility (shift from down to high food source) is still expected in *D* → *H* condition. Indeed, the strong individual preference for the ascending path compared to the descending one, observed for both species, must allow the flexibility independently from the colony size.

Additionally, it was shown that pheromone deposition towards a high-quality food source was higher than towards a low one^5, 44^. Previous studies provided evidence that symmetry breaking between two food sources was notably influenced by resource quality^51, 52^. In the case of two trails leading to high-quality food sources, both trails should be strongly marked while when two identical low-quality resources are available, individuals will mark a trail weakly or at the same level as ants that have not fed^53^. This phenomenon promotes high symmetry breaking when facing two low-quality sources, whereas when food quality was high, enhanced pheromone deposition on both branches caused a smaller relative difference in paths and weaker symmetry breaking, consistent with trail choice following Weber’s law^54, 55^. For a very low-quality sources and therefore very weak trail laying, it is intuitive to observe a weak symmetry breaking or a lack of asymmetry^48^. Our results in the symmetrical experiments did not reveal this kind of collective decision making. No difference in symmetry breaking occurred between the two non-preferred branches (*DD* condition) and the two preferred branches (*HH* condition).

Considering the well-known modulation of pheromone deposition in relation to food quality^5, 53^ (but not quantity^56^) and its consequences for recruitment intensity, we could expect more significant food collection when ants faced two preferred resources (like the *HH* condition) compared to two non-preferred ones (like the *DD* condition). However, our experimental results did not support this hypothesis, and foraging efficiency did not seem to be affected, as ants did not forage less when facing two non-preferred paths rather than two preferred paths.

To go one step further in understanding collective choice in the case of symmetrical and asymmetrical branches, we investigated the behaviour of foragers at the individual level. The relief of the branches seemed to be a determining factor leading to a clear collective choice in the case of asymmetrical *DH* condition in both studied species, but no information was available here to discuss the impact of this factor on the modulation of pheromone deposition at the individual level. In addition, although previous work showed the effect the of gravity on forager movement^51^ and travel speed^36, 40, 57^, we were not able to highlight an effect of slope (ascending or descending) or nutritional status (empty or full crop) on the average moving speed as no difference was revealed whether on back or forth travel on either branch; the average speed remained constant.

In the *DH* condition, within the first few minutes of the experiments and well before any pheromone deposition^10, 58^, the foragers show a preference for the ascending branch. At this stage, trail pheromones can be ruled out as the cause of this preference. We therefore investigated the behaviour of foragers arriving at the Y-maze during the first minutes for all conditions. An alternative mechanism for path selection, for which we found strong support, is U-turning behaviour. In the *DH* and *D* → *H* conditions, far more U-turns were performed by foragers facing a descending slope during the period of exploration (approximately 48%) than when facing an ascending slope (approximately 8%). Ants on their first nestward trip performed more U-turns on the descending path and were more likely to switch paths if they initially entered the descending path. It is perhaps not surprising that U-turns play a key role in path selection due to perceived path use, as they have been shown to play a key role in several collective decision mechanisms in ants^10, 44, 59, 60^. Although one information source may begin the decision-making process, other information sources may cause a decision to be maintained. In the *DH* condition, the initialization of colony-level path choice appeared to be based on individual preference via the U-turning mechanism. This former branch choice was then amplified by two other mechanisms: The first is pheromone deposition, which began a positive feedback cycle, with more ants choosing the ascending path because it had higher trail pheromone levels and therefore depositing more pheromones on this path^61^. The second mechanism that was likely to amplify the initial path choice pattern was route memory^62^, as it is well known that ants that took a particular path and were rewarded are likely to take the same path in the future^63–65^.

However, these mechanisms could not explain the observed switch in foraging activity in both species when the highest food source was introduced incidentally (*D* → *H* condition). The U-turn rate measured during the period of recruitment in both species supported the phenomenon of the switch of sources: Indeed, even if the pheromone trail was strong in the *D* → *H* condition towards the down food source at 20 min (as it was the only food source available during the first 20 min of the experiment), the U-turn rate of foragers facing the branch towards the down food source was higher (approximately 41%) than the U-turn rate of foragers facing the ascendant branch (approximately 2%). The results of the U-turn rates in the *DD* and *HH* conditions in both species also confirmed the individual preference for the ascending path. Indeed, in *DD*, the U-turn rate was high and equal between the two descending branches during both the exploration and recruitment phases. In contrast, the U-turn rate in the *HH* condition were surprisingly low in both the exploration and recruitment phases. These results suggest that ants could use different cues during food recruitment and not only the single signal of a pheromone trail, allowing ants to not get stuck in a sub-optimal solution in cases of a stronger pheromone trail towards a poorer food source. Undoubtedly, there are many ways for ant colonies to achieve flexibility in their recruitment^20, 66–68^ and different signals are often used to modulate recruitment (e.g., invitation behaviour and/or multi- component trail pheromones^69–72^). Substantial differences in the U-turn rate when facing ascending or descending paths seems here to play a central role in the flexibility of collective decision. This is supported by preliminary work on a theorethical model where the parameter of U-turns allows the colony to switch or not (in prep.). U-turns are already known to contribute to collective choice and trail strength in ants^44, 73^, but the mechanisms underlying the phenomenon of source switching still require further investigations, notably concerning the potential link between U-turn behaviour in relation to slopes and the modulation of pheromone deposition.

What are the ultimate reasons for the preference of ants to exploit the highest food source? The first hypothesis of energy savings is not so straightforward, as a recent study does not support this hypothesis. In their study, they highlighted that energy expenditure per unit distance was minimal on horizontal ground (slope of 0 deg) and significantly increased with an increasing gradient both on an ascending and a descending slope in leaf-cutter ants, *Acromymrex octospinosus* ^40^. This was clearly intriguing as, in our experiments, both species clearly preferred the ascending slope. The second hypothesis is an ecological hypothesis. *L*. *niger* feeds on the honeydew of aphids, such as *Tuberolachnus salignus* ^74^, *Aphis fabae* ^75^ or *Metopeurum fuscoviride* ^76^, which occur on the branches of trees while its nests are at the ground level. *M*. *rubra*, despite being myrmecochorous^77, 78^ and carnivorous^79^, also consumes sugar sources^80^, such as extra-floral nectar or honeydew^81^ on trees and bushes. The validation of the ecological importance of these laboratory results and hypotheses obviously needs field experiments.

Collective choices are largely based on the competition between positive feedbacks^47^. Each of them is characterized by its own rate of amplification which depends on behavioural modulation related to food characteristics^51^ or environmental/physical constraints^66, 82^. However these constraints (e.g. distance nest-food) may also affect the rate of recruitment without any behavioural modulation. Here we show another environmental variable that affects the collective decision: the relief. Moreover negative feedback acting upstream of the recruitments (such as satiety) or being a by-product of the foraging activity (such as food exhaustion) are also involved. These negative feedbacks can prevent the symmetry-breaking or facilitate the flexibility of collective response. The increase of the complexity of the recruitment mechanisms (multiple pheromones^66, 68^, leadership^24^) leading to an increase in the number of feedbacks also facilitate this flexibility. However, in our case such supplemental feedbacks and complexity do not seem necessary to produce flexibility.

Few studies have been conducted on land shape and its influence on foraging behaviour^57, 83^. Further investigations will be needed to determine how the relief affects the response of large colonies or the importance of the relief of a path to a food source compared with food source quality in the collective choice of ants by combining both determining factors into a single experiment, for example, by introducing a poor food source (0.1 M) at the end of a ascending branch and a rich one (1 M) at the end of descending branch. Our work was a first step towards better understanding the effect of relief of the environment on food source preference in ants and confirmed the large range of parameters taken into account in the collective choice of ants.

## Methods

### Study species

From ten large mother colonies of the black garden ant, *L*. *niger* (collected in Brussels, Belgium, Sept. 2015), and the red ant, *M*. *rubra* (Collected in Falisolle, Belgium, Sept. 2015), we created ten queenless and broodless subcolonies of 100 randomly chosen workers of each species (*L*. *niger* = *L100*; *M*. *rubra* = *M100*). An mature colony of L. niger can contain more than 5000 individuals. But due to the physical characteristics (number of chambers, number of nest holes) of the colonies not all the individuals forage in the same area. Moreover, young colonies contain only a few dozens and later few hundred workers. Colonies of around 100 individuals exhibit efficient trail recruitment^49^. The mature colonies of Myrmica rubra are smaller (e.g. the size of 60% of the colonies is less than 1000 individuals)^84^. Therefore, one hundred workers is a consistent number for both species. Colonies were maintained in plastic boxes (26 × 16 × 5 cm high) coated with Fluon® and contained a square glass nest (8 × 8 × 0.2 cm high) with a unique 3 mm wide entry. Colonies had access to water and a sucrose solution (0.3 M) ad libitum and were maintained at 21 °C ± 1 C and 60% ± 5 relative humidity with a constant photo-period of 12 hr per day. Before each experiment, we starved the colonies for 4 days to enhance food collection. Each colony was randomly tested in four of the five conditions with a 7 -day break between two consecutive trials.

### Experimental Design

**Figure 4:**
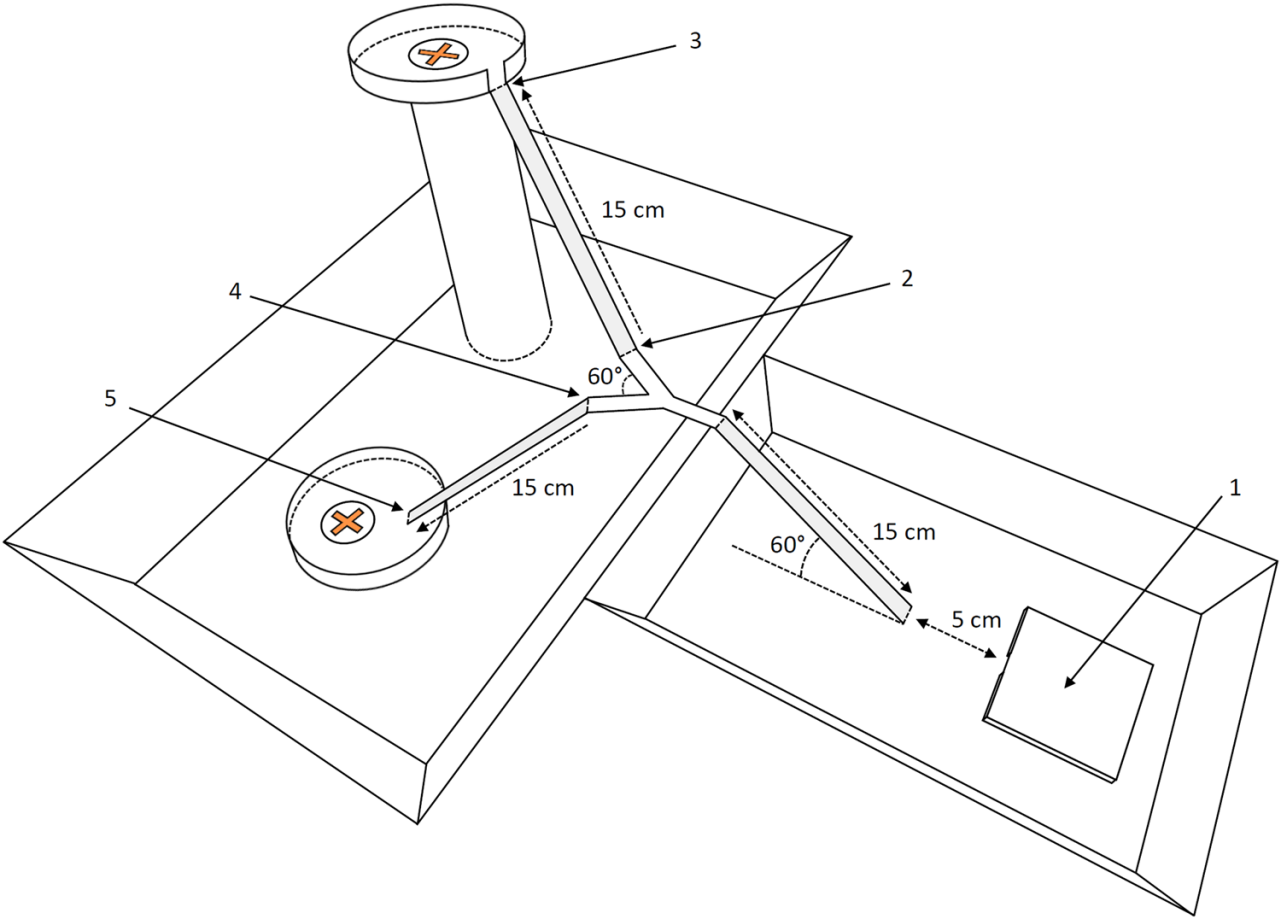
*HD* experimental setup. The nest (1) and its unique entry (2) facing the first branch of 15 cm leading to the T-maze with ascending and descending branches (*HD* setup shown). A syrup feeder (1 M sucrose) was placed on a platform at the end of either the ascending or descending branch of the maze. The number of ants was counted at each food source, and U-turns were measured at point (2) of the ascending branch and (4) for the descending one.

Each colony was introduced to the experimental setup 30 min before a trial in a closed environment with blank walls and lighted with homogenous diffused light. After 4 days of starvation, the ants were provided access to two food sources of 1 M sucrose first through a rising slope (15 cm long and 10 cm high, see (1) in Fig. 4) introduced 5 cm from the nest. This first slope led to an asymmetric Y-maze with two same-sized branches separated by a 60 angle. Liquid sucrose was introduced to a circular plastic cup placed in the centre of an 8 cm diameter Petri dish at the end of each branch. The two food sources strictly contained strictly the same concentration (1 M) and volume (3 ml) of liquid sucrose.

We tested five different experimental conditions:

- *Down-High Simultaneous (DH)*: Two food sources that ants were freely available to exploit were simultaneously introduced. Each branch was characterized by its slope: the right branch was ascending (15 cm long by 10 cm high), leading to the upper food source, while the left one was descending (15 cm long by 10 cm down), leading to the lower food source (*L*. *niger*: N = 9, *M*. *rubra*: N = 9).
- *High-Down Simultaneous (HD)*: This condition mirrors that of *DH*: the right branch ascending to a food source and the left one descending (L. niger: N = 9, *M*. *rubra*: N = 9).
- *Down-High Lag (D* → *H)*: The aim of this condition was to test the ability of the ants to reallocate the forager workforce towards a new food source when it was introduced 20 min after the first one. At the beginning of the experiment, ants had access to both branches, but only the down food source was filled with liquid sucrose, and ants started to exploit it. After 20 min, the high food source was filled (*L*. *niger*: N = 7, *M*. *rubra*: N = 7).
  Two symmetrical conditions were also tested:
- High-High Simultaneous (HH): In this condition, the two branches of the Y-maze were ascending and the two food sources were simultaneously introduced (L. niger: N = 6, M. rubra: N = 7).
- Down-Down Simultaneous (DD): In this condition, the two branches of the Y-Maze were descending and the two food sources were also introduced simultaneously (L. niger: N = 7, M. rubra: N = 7).

### Data Collection and Analysis

Video data were recorded using a Sony DMC-GH4-R mounted with a 12 mm lens capturing 25 frames/s at the definition of 4180 × 2160p. We investigated the ants’ behaviour at two levels:

(i) The individual level: We manually measured the time spent by foragers feeding on each of the two food sources, the time needed to cross a branch (Fig. 4) (between (2) and (3) for the ascending branch and between (4) and (5) for the descending one, distance = 15 cm), and the number of U-turns (i.e., ants returning to where they came from without exceeding half of the branch instead of continuing their path) on each branch in both the Nest → Source and Source → Nest direction. The time needed to cross branches and the U-turn rate were measured in all experimental conditions, during the first 5 min of experiments with *L*. *niger* and the first 10 min for *M*. *rubra*. This first period of observation was called the “exploration” phase, as few or no pheromone trails had been deposited on each branch because no or only a few foragers had gone back to the nest after having exploited the food source at the beginning of the experiment. U-turns were also measured in all conditions (except *DH*), respectively, for 20 to 25 min for *L*. *niger* and for 20 to 30 min for *M*. *rubra*. This was named the “recruitment” phase, as it corresponded to the average peak of foraging activity (maximum number of foragers simultaneously exploiting food sources) in our experiments. U-turns in the *DH* condition were not measured during “recruitment”, as choice occurred rapidly towards one source in this asymmetrical setup and almost no ants used the second branch. The measurement period for *M*. *rubra* was twice as long as for *L*. *niger* due to the slower speed of travel and the longer time spent at food sources in *M*. *rubra*; a longer period of observation was requested to achieve the same sample size.

(ii) The collective level: An open-source tracking software (USE Tracker: http://usetracker.org/) was used to automatically estimate the number of ants feeding at each food source in each frame of the video. We assumed that an immobile ant with its head and antennae above the liquid sucrose was collecting food. Thus, the time spent by each forager at a food source was used as a proxy for the amount of food collected. From these data we generated the cumulative number of ants at each food source, defined as the overall effort of food collection. Because of the variability in the detected number of entities in each frame, we averaged this number over a range of 10 sec (see Supplementary Figs S1–S4 for details on the ant tracking procedure). We applied a binomial test on the cumulative number of presences at each food source at the end of the experiments to determine significant difference between relative exploitation of both food sources. For the D → H, this test was also applied to determine the choice 20 min after experiment began. Henceforth, a significant choice oriented first towards the lower food source for 20 min then significantly towards the highest food source at the end of the experiment was considered to be a “switch”. Thus, the recorded parameters were the number of ants simultaneously present on each food source during the entire experiment as well as the time of maximum simultaneous presences and the moment when a choice occurred (last crossing of the two curves). The time of first discovery of each food source was also manually recorded. Filming began when the bridge was placed in front of the nest and ended 2 hr later.

Automated processing of all experiments was performed with Python 3.5.1, statistical analyses were conducted with SciPy 0.17.0, and visualization of the data sets was done with Matplotlib 1.5.1. Our data were not normally distributed. We used a linear model to test correlation. The multiple comparison Kruskal-Wallis test coupled to Dunn’s post hoc comparison of pairs was used to compare conditions. The Mann-Whitney U-test was used in cases where we had only two groups to compare. As advocated by Oron and Hoff ^85^ when data set needs a tie correction and parametric assumption is violated, we used permutation Kruskall-Wallis tests to analyse nested effects in our hierarchical design. We checked the homogeneity of colonies’ responses by carrying out Kruskal-Wallis permutation tests that compared observed data distributions with randomised data distributions (N = 1000). Concerning relative exploitation of the two food sources, colonies displayed similar collective behaviour (permutation Kruskal-Wallis tests: *M*. *rubra*: H = 0.77, P = 0.83; *L*. *niger*: H = 0.70, P = 0.78). The differences were considered to be significant at *P* < 0.05 for all tests.

## Electronic supplementary material

Supplementary
