# Supplementary material for: Effect of the land area elevation on the collective choice in ants
Source: Sci Rep. 2017 Aug 18;7:8745. doi: 10.1038/s41598-017-08592-9 (PMC5562813; doi:10.1038/s41598-017-08592-9)
Supplement: Supplementary file 1 — Supplementary [file 41598_2017_8592_MOESM1_ESM.pdf]

Effect of the land area elevation on the collective choice in ants.

Olivier BLES, Nathanaël Lozet, Jean-Christophe de Biseau, Alexandre Campo, Jean-Louis Deneubourg

Supplementary information

S1. Automatic counting of ants feeding at a food source.

Initial processing of the data using USETracker software, ants were detected by background subtraction algorithm on each frame (25 frames/sec) of the 2 hr movies of the experimental setup (Figure S1 B). Numerical output for each frame is the total number of pixels detected at each food source, corresponding to feeding ants. Values collected on each frame were averaged on a 10 seconds basis to reduce the effect of noise in the total number of detected pixel, $P_{detected}(t)$ = mean number of pixels detected in 250 consecutive frames (10 sec x 25 frames/sec). We calculated the parameter *Size* = mean size in pixels of a single detected ant for both species (as *M. rubra* is larger than *L. niger*) at each food source (high and down). Indeed the camera was vertically placed above the setup and the high food source was nearest to camera than the down food source. Therefore a forager occupied a greater surface on the movie when it was at the high food than when it was at the down food source (Figure S2). We then estimated the number of ants at each food source at each time step of 10 sec, N_{mean}(t), during 2 hr of experiment, by dividing the mean number of detected pixels during 10 secs by the mean size of a single detected ant: $N_{mean}(t) = P_{detected}(t) \,/\, Size$

The accuracy of estimation by the USETracker was then assessed by manually counting the number of ants simultaneously feeding at the food source every minute during the 2 h of an experiment (Figure S3). The coefficient of correlation was around 0.99. and we can see only few times during the 2 h of experiment where the manually counted value and the automatic estimation from the USETracker software were different (never over 1 point).

Once the accuracy of the USETracker was validated, we generated the cumulative number of ants at each food source with the data from USETracker for all the experiments (*e.g.*, Figure S4, S5, S6).

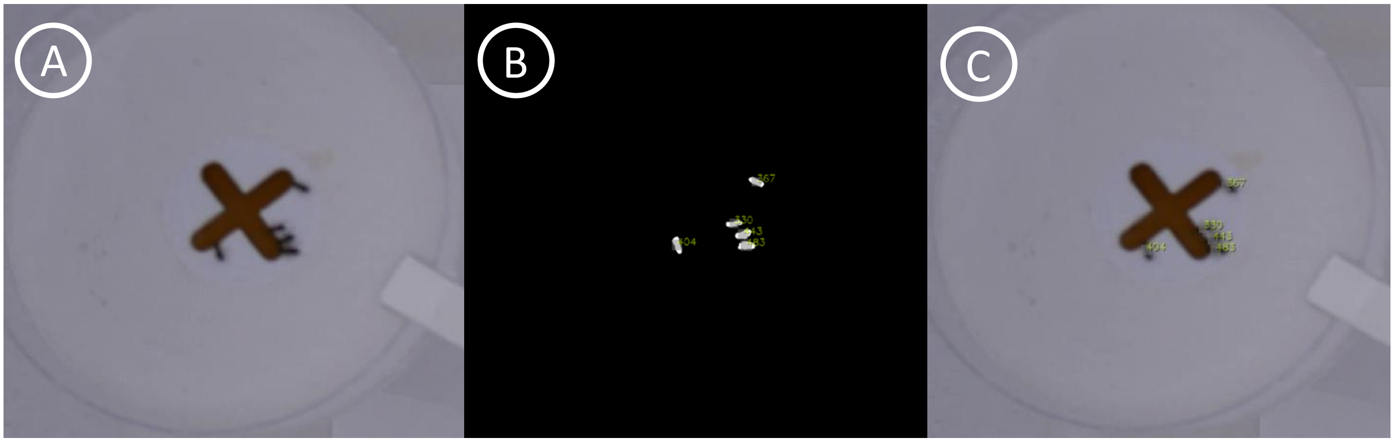

Figure S1. Ants of *L. niger* feeding at a food source are automatically detected by USETracker software. **A** Input frame. **B** Processing frame. **C** Output frame.

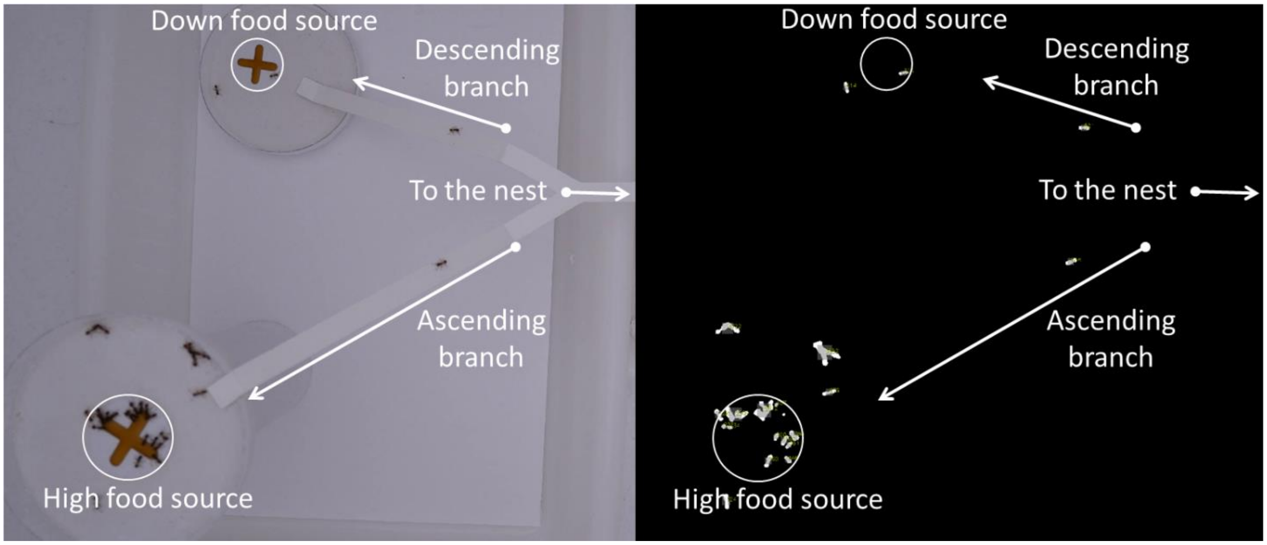

Figure S2. Ants of *M. rubra* of a *HD* experiment feeding at the high and down food sources from **A** a screenshot of the movie and **B** same frame processed by USETracker.

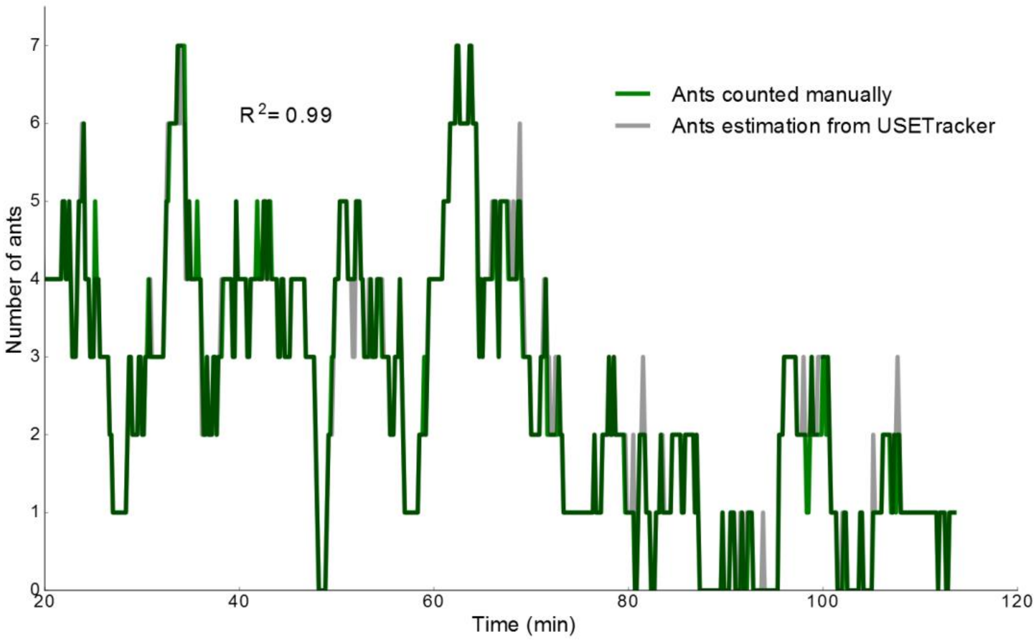

Figure S3. Instantaneous number of ants of *L. niger* of a *HD* experiment feeding at a food source from manual counting (green line, timestep = 1 min) and automatically counted by USETracker (grey line, timestep = 10 sec) during 2 h of an experiment.

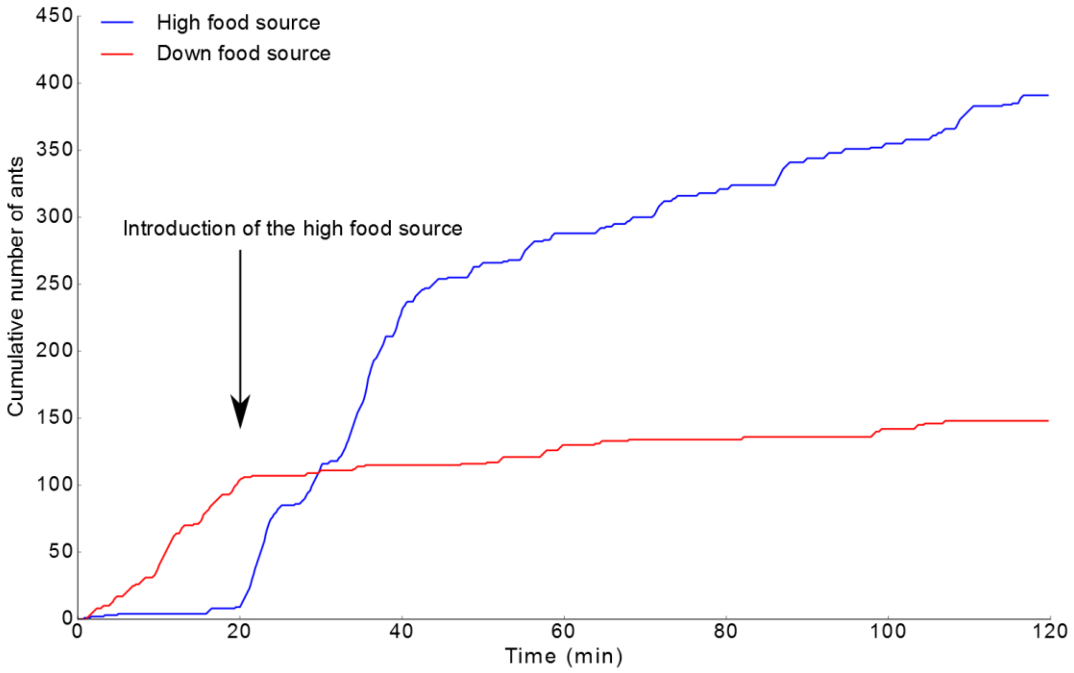

Figure S4. Exemple of cumulative number of ants of *M. rubra* of a *High*→*Down* experiment feeding at the high and the down food source automatically estimated by USETracker during 2h. The high food source was introduced at 20 min. The fast and important exploitation of the high food source from 20 min is clearly apparent. This two curves illustrate a switch of food source exploitation during the experiment (see Methods and Results sections for more explication on this result).

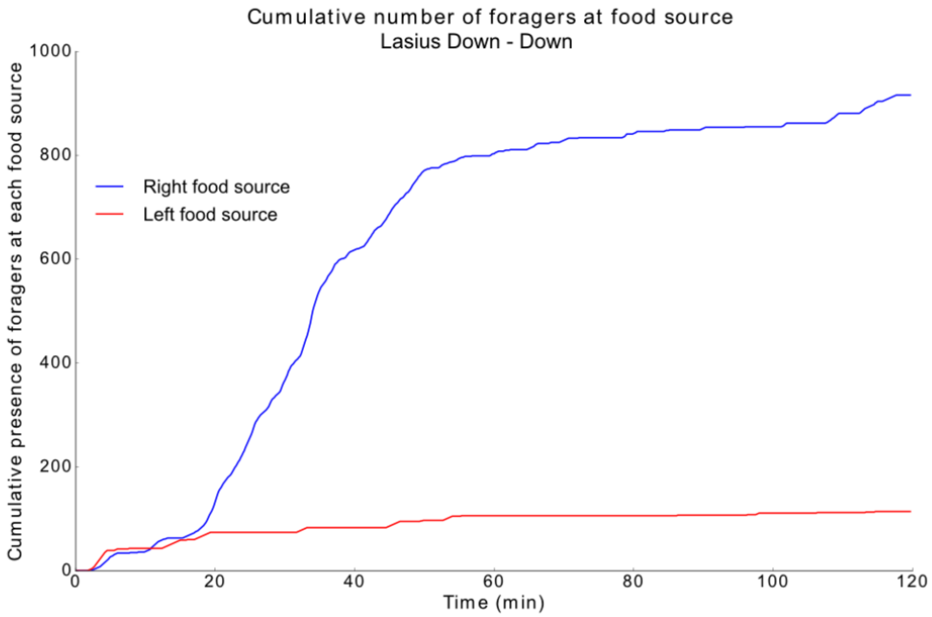

Figure S5. Exemple of cumulative number of ants of *L. niger* of a *Down - Down* experiment feeding at the left and the right food source automatically estimated by USETracker during 2h. The two food sources were simultaneously introduced at the beginning og the experiment. A first phase can be identified, with the arrival of the scouts at food until 20 min before recrutment occurred after 20 min with numerous simulataneous arrivals of new foragers to food source. This two curves illustrate a symmetry breaking between the two symmetrical branches.

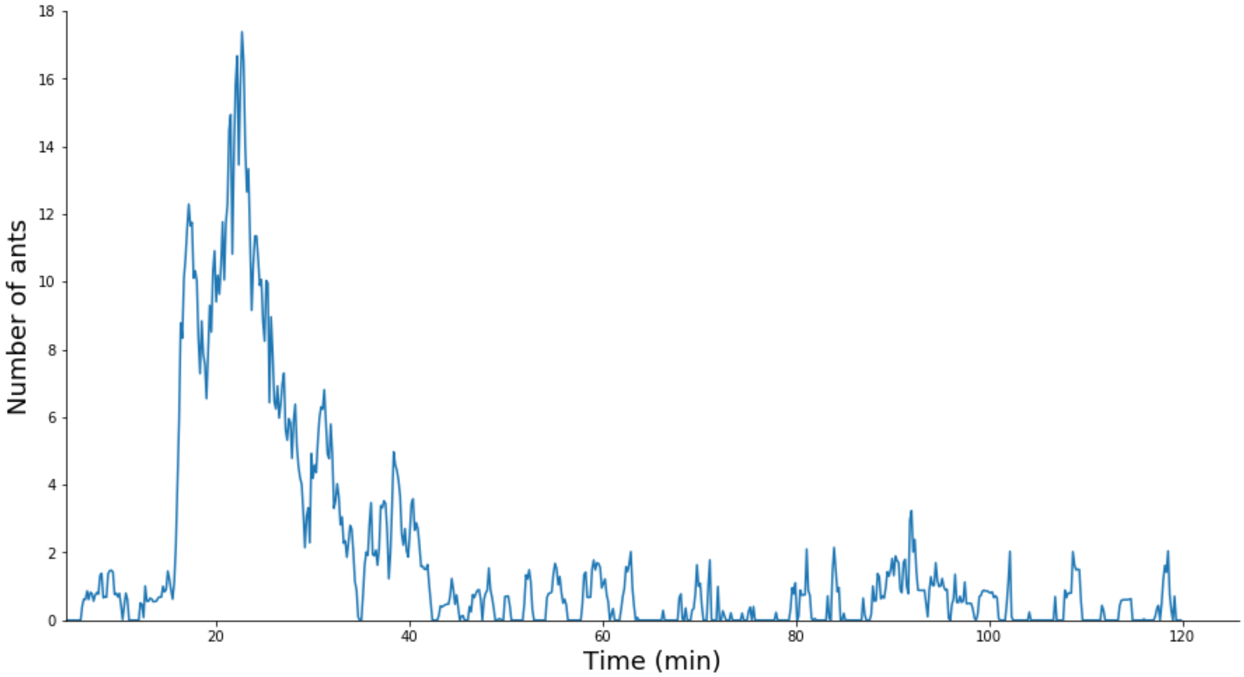

Figure S6. Total number of foragers simultaneously feeding at the two food sources during 2 hours in a colony of 100 ants of *L. niger* in a *DH* experiment. The effect of recrutment lead to an important flow of foragers at food sources around 15 min, with a peak of foraging activity around 25 min before colony satation and a decreasing number of foragers feeding at food sources happened around 40 min. The duration of experiment was chosen to allow the colony to achieve satiety.
